# Supplementary material for: Identification of Growth-Related Gene BAMBI and Analysis of Gene Structure and Function in the Pacific White Shrimp Litopenaeus vannamei
Source: Animals (Basel). 2024 Apr 1;14(7):1074. doi: 10.3390/ani14071074 (PMC11011141; doi:10.3390/ani14071074)
Supplement: Supplementary file 1 [file animals-14-01074-s001.zip › Supplement of LvBAMBI/Data 1.pdf]

**>LvBAMBI**

**ATG**GACTTTACGCTCAAGGAAGTTTGGCGACGGACATTATGTTGCCTCGTGTCCCT
CGTCATCTTACAGCTCCTGGGAGACACAGTGTCGGTCGCTAATGGAGAATTTTTGC
ACAAGAACGACACCAACATTAGCGAAGATTCGACAGCGGCGATCATGGACAAA
GAAGCGCCAGAGGAGAATAACGAAGGAGGCGAAGTCCGGTGCCACTGCAACCT
CCCTCGGTGCGTCACAGTAGGCTACATGTGTAAGAGCTCCCTGGGTGCCTGCTTC
ACGCGCCCCACGCCCATGCCCACGCCCGCGCCCGCCACCCGGAGGCTCTACCAG
CGGAGGACGCCCACGCACGGCTGTTTGGAGCTTTTGCCCGAAGACCGGCATGCA
GAATGTGCTACCAAGGCTGGGGTCTCAAACAAGAGCATTATATTGAGGTCACAGA
AAACATCAGCAAAGATGGTAGAAACTACTTCAAACAGTGCCTCGCGTCCTACTCC
TTCTGCGAGTCCTACCCTTCCTCCAGCTGACCCCAATCTGACATGCTGTAGCCAGG
ATATGTGCAATTATCGAGACCTGGATGTCTTCATAAGGGTAGACAAGGCTAATACC
CAGAAAGATTACAGCAGAGGAGAATCAGAAATGCTGGAGACAGTGTGGTTCCGA
GCAGCTACCATTGCAGTGCCCATAGCAGGGGGTTTCATCCTCATTGTGTTGGTCTT
CCTGGCTAGTAGAATGCTGGCCAAGGAAAACAAGAGGCAGAGGATGGCTCAGGT
GATTAATGAGCGGTACCTGAAGGCTCCCCTGTATCCTGGGGGCTGTGCAGCTGAG
CCTTTGCCACCACCACAGCATTCTCTCTACTACCACCCACCACTGCTCAAGAACAT
CTCACTGGGCCATGTCAATCTGCGGGCATCATATGAGGACAAGCCGCCTAGCTAC
CGCTCCCCAGGGATGCATGTCTGCAGCCTGGAGGAAATCCGACCCCTGAATGGG
GTGATGGGCATGCCAGATGGGGGAGGAGGAGGATTACAGGGAGGGGTGCACTGG
GACCAGAGAGGAGCTGAGTCGCCTCTTCCGTCA**TGA**

**Protein sequence**

MDFTLKEVWRRTLCCLVSLVILQLLGDTVSVANGEFLHKNDTNISEDSTAAIMDKEAPE
ENNEGGEVRCHCNLPRCVTVGYMCKSSLGACFTRPTPMPTPAPATRRLYQRRTPTHG
CLELLPEDRHAECATKAGVSNKSIILRSQKTSAKMVETTSNSASRPTPSASPTLPPADPN
LTCCSQDMCNYRDLDVFIRVDKANTQKDYSRGESEMLETVWFRAATIAVPIAGGFILIV
LVFLASRMLAKENKRQRMAQVINERYLKAPLYPGGCAAEPLPPPQHSLYYHPPLLKNI
SLGHVNLRASYEDKPPSYRSPGMHVCSLEEIRPLNGVMGMPDGGGGGLQGGVHWD
QRGAESPLPS
