# Supplementary material for: Identification of Growth-Related Gene BAMBI and Analysis of Gene Structure and Function in the Pacific White Shrimp Litopenaeus vannamei
Source: Animals (Basel). 2024 Apr 1;14(7):1074. doi: 10.3390/ani14071074 (PMC11011141; doi:10.3390/ani14071074)
Supplement: Supplementary file 1 [file animals-14-01074-s001.zip › Supplement of LvBAMBI/Figures S1, S2.pdf]

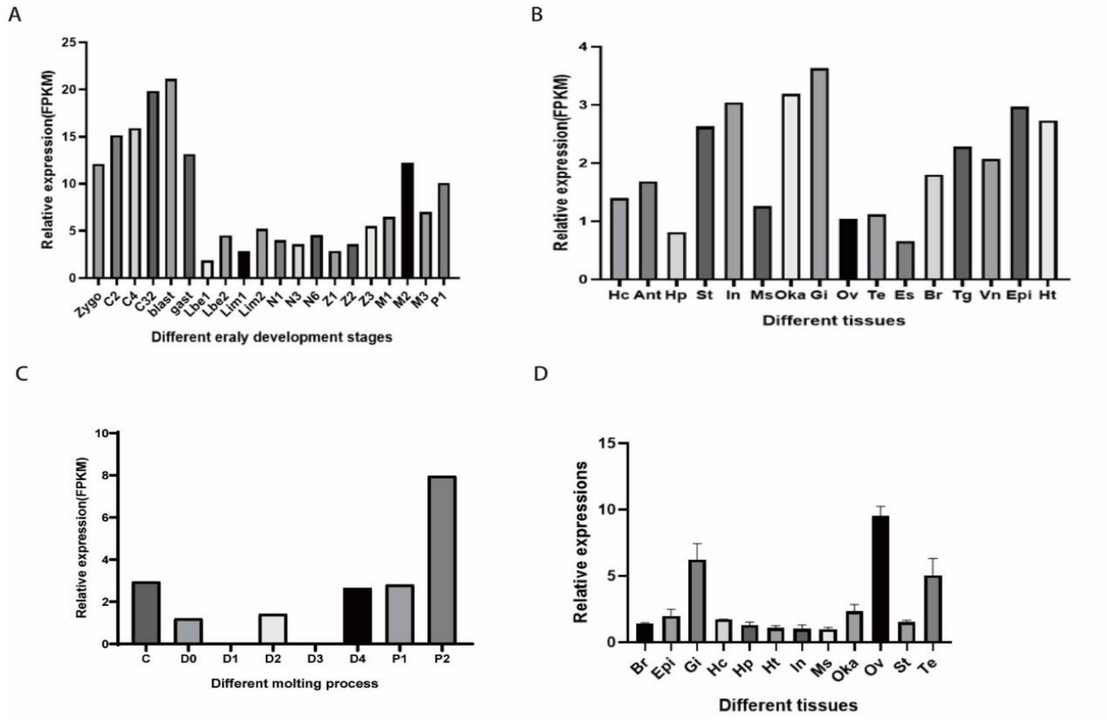

Figure S1. The expression profiles of *LvBAMBI* gene. (**A**) Early development stages: zygote (zygo), 2 cells (C2), 4 cells (C4), 32 cells (C32), blastula (blast), gastrula(gast), limb bud embryo I (Lbe1), limb bud embryo II (Lbe2), larva in membrane I (Lim1), larva in membrane II (Lim2), nauplius I (N1), nauplius III (N3), nauplius VI (N6),zoea I (Z1), zoea II (Z2), zoea III (Z3), mysis I (M1), mysis II (M2), mysis III (M3), and postlarvae 1 (P1); (**B**) adult tissues: Hc, hemocyte; Ant, antenna; Hp, hepatopancreas; St, stomach; In, intestines; Ms, muscle; Oka, lymphoid organ; Gi, gill; Ov, ovary; Te, testis; Es, eye stalk; Br, brain; Tg, thoracic ganglion; Vn, ventral nerve; Epi, epidermis; and Ht, heart. (**C**) molting stages: intermolting (C), premolting (D0, D1, D2, D3, and D4), and postmolting (P1 and P2) stages; (**D**) The relative expression levels of LvBAMBI in the tissues of 12 adult shrimp species were quantified using qPCR with the gene expression level of 18S as a control.

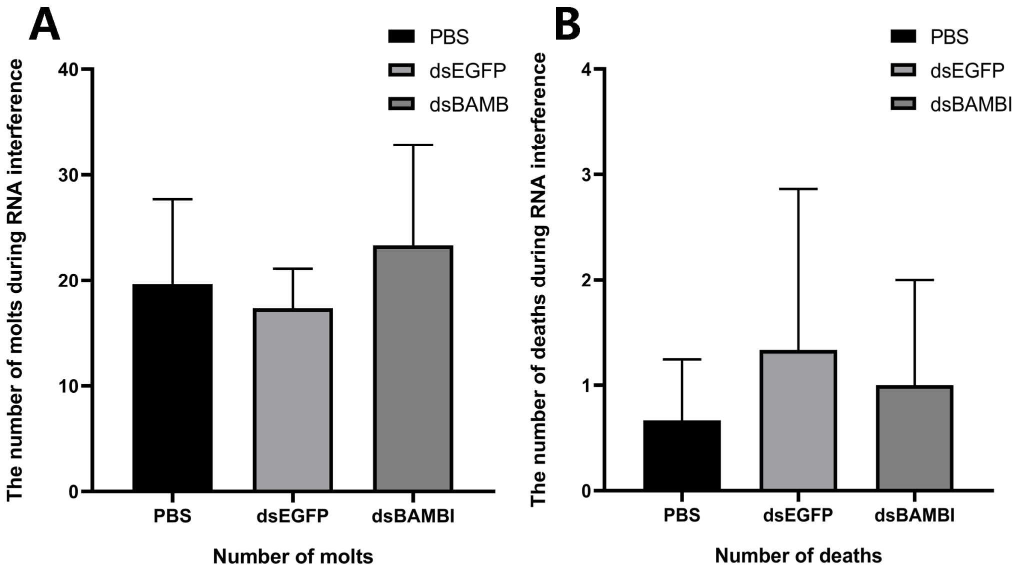

Figure S2. Counts of number of molting and death between control and experimental groups after RNA interference of *LvBAMBI*.
